# Treatment of Stomatitis Materna

**Published:** 1878-07

**Authors:** E. P. Hurd

**Affiliations:** Newburyport, Mass.


### ARTICLE X.

### *Treatment of Stomatitis Materna.*

BY E. P. HURD, M. D., NEWBURYPORT, MASS.

The pathology of this affection, which is sufficiently common in the experience of general practitioners, is obscure; is seen in patients who seem to be well in other respects; is generally an index of lowered vitality: sometimes an inflammation, the result of local nutritive disturbances of reflex origin, the irritation being mammary. In the ptyalism of pregnancy we see an instance of the same etiological kind:

Stomatitis materna, takes the form of acute or chronic oral catarrh, also aphthous and ulcerative stomatitis.

It is sometimes very obstinate and intractable. Weaning the child always brings about a speedy cure. It is, however, generally amenable to treatment of a simple nature. Where from lowered vitality, a tonic regimen, as quinine, porter, iron and cod-liver oil is required, there are two preparations of the latter in the market, which I find almost equally serviceable—Scott's Emulsion and Phillips'. Many, however, can take Phillips' oil that cannot take Scott's. Phillips' oil is borne by the most delicate stomachs. I consider this preparation invaluable for nursing women whose nutrition is below par.

Nursing sore-mouth will often yield to astringent gargles, or mouth-washes. Chlorate of potash is good in this affection, as in all other oral affections of an inflammatory and ulcerative nature.

The glycerite of tannin, a great favorite of Ringer in all these complaints (see his *Handbook of Therapeutics*, page 300,) is sometimes all the application that is needed—to be brushed over the parts affected twice or three times a day Where astringents alone will cure, this will cure.

The sulphites, especially the sulphite of sodium, the sulpho-carbolate of sodium, are recommended. The sulphite of sodium may be given internally, in doses of a scruple three times a day, and also used as a mouth-wash. The same dose (1 scruple) may be given of the sulpho-carbolate of sodium, and, dissolved in an ounce of water, used as a gargle. These medicines undoubtedly have an alterative effect, as well as a local action (see Braithwaite, Part 63, page 24.)

The tincture of smartweed (*Polygonum punctatum*) in drachm doses, diluted with water, to be taken internally, and used as a gargle, is highly recommended by a writer in the *Philadelphia Med. and Surg. Reporter.* Another authority regards bismuth, in ten-grain doses, as a certain remedy.

In several obstinate cases I have used the following formula with good results. (The formula is borrowed from the *Reporter.*) In one case cure followed in less than a week, but during its use the baby, previously well, was severely griped and purged. I think that it should be used with much caution, and discontinued in a few days if it does not seem to do good, or if it affects the baby injuriously. The dose of the biniodide is about one thirtieth of a grain:

℞. Hydrarg. biniod, gr. v. Pot. iod. gr. x. Aquæ ℥j. M. Ft. sol. liq.

Take three to five drops, after meals. For a mouth-wash, add six drops to a tablespoonful of water.

This remedy, besides its local stimulant and resolvent action, undoubtedly does good as an alterative, improving local and general nutrition.—*Med. Record.*

